# Deletion of the γ-secretase subunits *Aph1B/C* impairs memory and worsens the deficits of knock-in mice modeling the Alzheimer-like familial Danish dementia

**DOI:** 10.18632/oncotarget.7389

**Published:** 2016-03-01

**Authors:** Fabrizio Biundo, Keita Ishiwari, Dolores Del Prete, Luciano D'Adamio

**Affiliations:** ^1^ Department of Microbiology and Immunology, Albert Einstein College of Medicine, Bronx, New York, United States of America; ^2^ Research Institute on Addictions University at Buffalo, Buffalo, New York, United States of America

**Keywords:** APP, Itm2b-bri2, Alzheimer, Danish dementia, gamma-secretase, Gerotarget

## Abstract

Mutations in *BRI2/ITM2b* genes cause Familial British and Danish Dementias (FBD and FDD), which are pathogenically similar to Familial Alzheimer Disease (FAD). BRI2 inhibits processing of Amyloid precursor protein (APP), a protein involved in FAD pathogenesis. Accumulation of a carboxyl-terminal APP metabolite –β-CTF- causes memory deficits in a knock-in mouse model of FDD, called FDD_KI_. We have investigated further the pathogenic function of β-CTF studying the effect of *Aph1B/C* deletion on FDD_KI_ mice. This strategy is based on the evidence that deletion of Aph1B/C proteins, which are components of the γ-secretase that cleaves β-CTF, results in stabilization of β-CTF and a reduction of Aβ. We found that both the FDD mutation and the Aph1B/C deficiency mildly interfered with spatial long term memory, spatial working/short-term memory and long-term contextual fear memory. In addition, the Aph1BC deficiency induced deficits in long-term cued fear memory. Moreover, the two mutations have additive adverse effects as they compromise the accuracy of spatial long-term memory and induce spatial memory retention deficits in young mice. Overall, the data are consistent with a role for β-CTF in the genesis of memory deficits.

## INTRODUCTION

APP plays a central role in the pathogenesis of both sporadic and familial AD. Indeed, *APP* mutations that alter APP processing either protect from sporadic AD or cause familial AD; additionally, mutations in genes that regulate APP processing -such as *PSENs* and *BRI2/ITM2B*- cause FAD, FBD and FDD [1-12].

As briefly mentioned above, mutations in *BRI2/ITM2B* cause the AD-like autosomal dominant FBD and FDD [5, 7]. FBD is characterized by the early onset of personality changes, memory and cognitive deficits, spastic rigidity, and ataxia [5]. FDD patients present early onset cataracts, deafness, progressive ataxia and dementia [7]. BRI2 is a type II membrane protein of 266 amino acids that is cleaved at the C terminus into a peptide of 23 amino acids (Bri23) plus a membrane-bound mature BRI2 (mBRI2) product [13, 14]. In FBD patients, a point mutation at the stop codon of *BRI2* results in a read-through of the 3′-untranslated region and the synthesis of a BRI2 molecule containing 11 extra amino acids at the COOH terminus. Cleavage by convertases generates a normal mBRI2 plus a longer peptide, the ABri peptide. FDD is caused by a10- nucleotide duplication before the stop codon of the *BRI2* gene, which leads to the synthesis of a longer (277 amino acids) mutant protein [7, 15]. Convertase-mediated processing of the Danish mutant protein generates a longer C-terminal fragment, called ADan, and a normal mBRI2 polypeptide. Both ABri and ADan are deposited as amyloid fibrils. Of note, ADan deposits together with APP-derived Amyloid (Aβ42) peptides forming wide-spread amyloid angiopathy in the small blood vessels and capillaries of the cerebrum, choroid plexus, cerebellum, spinal cord, and retina [15]. Overall, FBD and FDD patients present cognitive dysfunctions and neuropathology including neurodegeneration, amyloid, and neurofibrillary tangles [7, 15-17], which are similar to those of Alzheimer's patients.

Knock-in mice models of FDD and FBD (FDD_KI_ and FBD_KI_ mice) showed that the mutant BRI2 proteins are mainly targeted for degradation, leading to a loss of mBRI2 function and, consequently, increased APP processing. Of note, loss on mBRI2 and increased APP processing was also detected in brain lysates from FDD and FBD patients. These alterations in APP processing, and not amyloid lesions, mediate memory and synaptic plasticity deficits caused by *BRI2/ITM2B* mutations. In fact, synaptic and memory deficits or FDD_KI_ mice were reduced by inhibition of β–cleavage of APP, which generates the fragments β-CTF and sAPPβ [18], while they were worsened by inhibition of γ-secretase, which cleaves β-CTF into Aβ and AID/AICD [6, 19-27].

These findings suggest that increases in β-CTF can be neurotoxic and predicts that reducing γ-cleavage of APP has pathogenic consequences, while enhancing clearance of β-CTF is therapeutically advantageous. Several data support the first hypothesis: 1) loss of γ-secretase in the mouse brain induces neurodegeneration, memory and synaptic plasticity deficits; 2) Presenilins mutations associated with FAD cause a loss of γ-secretase function [28-36]; 3) sub-chronic administration of GSIs impairs normal cognitive function in APP transgenic mice [37]; 4) the GSI Semagacestat exacerbated cognitive deficits and impaired activities of daily living in human AD patients [38].

The evidence that reduction of γ-secretase activity impairs cognitive functions is consistent with a negative effect of β-CTF. Yet, γ-secretase cleaves other type I trans-membrane proteins, including the APP-like Protein 1 and -2, Neuregulin-1 and Notch [39-44] and cognitive functions deficits prompted by γ-secretase inhibition could be caused by decreased processing of any combination of γ-secretase substrates.

γ-secretase is a multi-molecular complex comprising the catalytic subunits PSEN1 or PSEN2 and three accessory proteins: Anterior Pharynx-Defective 1 (Aph1), Nicastrin and Presenilin Enhancer Protein 2 (PEN2) [45-47]. Humans have two *APH1* genes (*APH1A* and *APH1B*) [48, 49]; rodents have three because of a duplication of *Aph1B* that gave rise to the *Aph1C* gene [50, 51]. Aph1A-containing γ-secretase complexes are essential for Notch processing, while APP and Neuregulin-1 are preferred substrates of γ-secretase complexes containing either Aph1B or Aph1C; hence, *Aph1BC^−/−^* mice show increased β-CTF but decreased Aβ peptide due to reduced γ-processing of β-CTF [50-53] and allow studying the consequence of inactivating γ-processing of APP limiting the confounding effects of inhibition of processing of other γ-secretase substrates.

Pharmacological evidence suggests a synaptic-toxicity of β-CTF. Here, we have tested this hypothesis genetically. If increases in β-CTF prompt learning and memory deficits, *Aph1BC^−/−^* mice may show deficits similar to those observed in FDD_KI_ mice; additionally, deletion of *Aph1BC* may worsen the defects of FDD_KI_ mice. On the contrary, if Danish mice develop learning and memory defects due to over-production of Aβ, the *Aph1BC^−/−^* mutation will ameliorate learning and memory deficits of FDD_KI_ mice.

## RESULTS

### Young mice carrying the FDD mutation and deletion of *Aph1B/C* show mild learning and memory deficits

**Figure 1:**
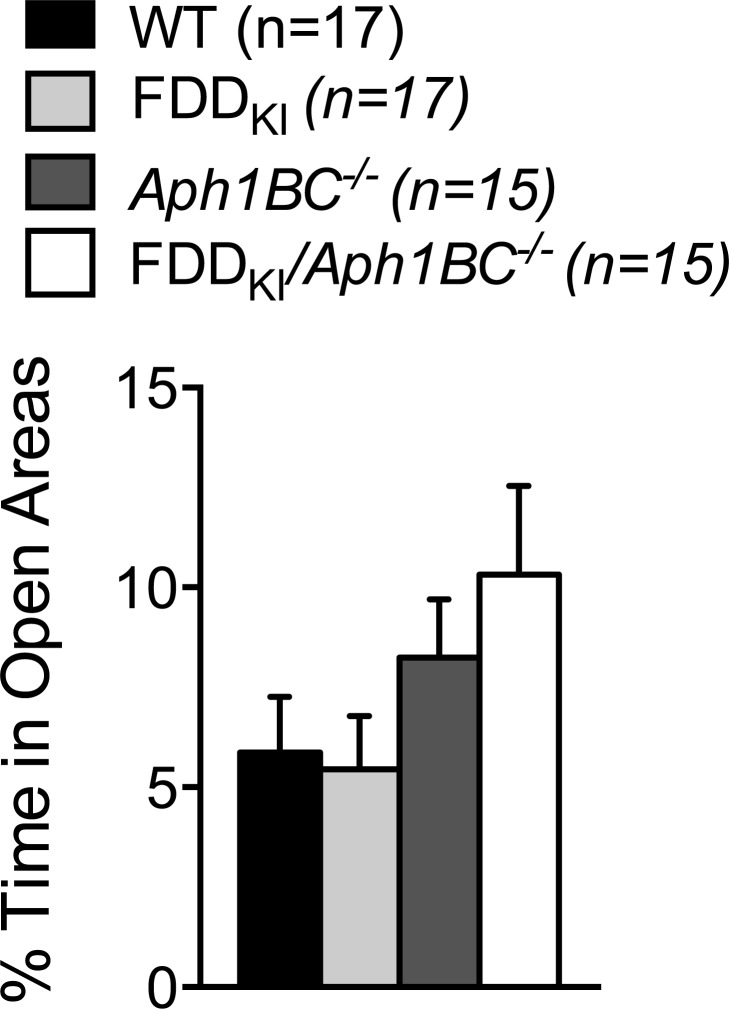
Elevated Zero Maze test on mice at 4 months of age Data are expressed as means ± S.E.M. No significant effect of genotype was found.

Mice were first tested at four months of age for anxiety-like behavior on the elevated rero maze. We analyzed the percentage of time spent in the open areas of the elevated zero maze during the 5-min testing period. While FDD_KI_*/Aph1BC^−/−^* mice spent more time in the open areas on average than mice of the other genotypes, one-way ANOVA revealed no significant effect of genotype, F(3, 60) = 1.94, *p* = 0.1331. Twelve animals (3 WT, 2 FDD_KI_, 4 FDD_KI_*/Aph1BC^−/−^*, and 3 *Aph1BC^−/−^*) fell off the open areas of the maze during testing and were excluded from the data analysis. In addition, two mice (1 FDD_KI_ and 1 FDD_KI_*/Aph1BC^−/−^*) were excluded due to technical problems with video tracking encountered during testing (Figure 1).

**Figure 2:**
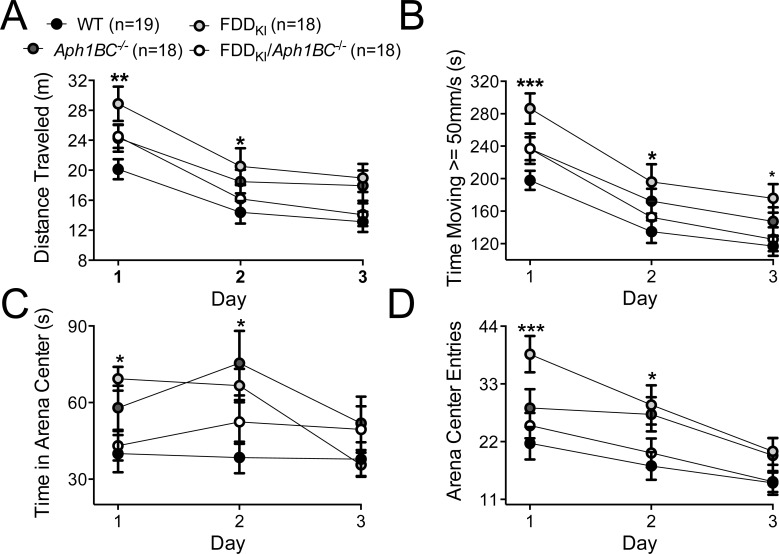
Open Field test on mice at 4 months of age Data are expressed as means (± S.E.M.) during the 10-min testing period over 3 days. **A.** Total distance traveled. **B.** Amount of time in which the animal ambulated at speed greater than 50 mm/s. FDD_KI_ mice were more active than WT mice, especially on Day 1. **C.** Amount of time the animal spent in the center of the arena (20 cm × 20 cm). **D.** Total number of entries into the arena center. FDD_KI_ mice entered and spent more time in the arena center on Days 1 and 2 than WT mice. * *p* < 0.05, ** *p* < 0.01, *** *p* < 0.001, WT *vs*. FDD_KI_.

Next, mice were assayed for general locomotor activity levels and anxiety-like behavior in the open field. Four mice (1 FDD_KI_ and 2 FDD_KI_*/Aph1BC^−/−^*) were dropped from the statistical analysis due to persistent tracking errors caused by their light-colored fur. Since the video tracking system was not able to track these mice consistently, they were not included in the subsequent experiments. In addition, one FDD_KI_ mouse was excluded from the analysis due to a one-time tracking error during testing. Analysis of the mean distance traveled during the 10-min testing period by two-way ANOVA found a significant main effect for day, F(2, 138) = 82.23, *p* < 0.0001, indicating habituation to the box over the three-day testing period, and a significant main effect for genotype, F(3, 69) = 3.46, *p* < 0.05, but no significant interaction between genotype and day, F(6, 138) = 1.15, *p* = 0.3387. *Post-hoc* comparisons (Dunnett's) showed that the mean distance traveled by FDD_KI_ animals were significantly greater than that traveled by WT mice on the first (*p*<0.01) and second (*p*<0.05) days (Figure 2A). Analysis of the time spent traveling at speed greater than 50 mm/s yielded significant main effects for day, F(2, 138) = 136.2, *p* < 0.0001, and for genotype, F(3, 69) = 3.97, *p* < 0.05, but no significant day × genotype interaction, F(6, 138) = 0.95, *p* = 0.4583, and showed that FDD_KI_ mice spent more time traveling at speed greater than 50 mm/s than WT mice on all three days (*p*<0.001, Day 1; *p*<0.05, Days 2 and 3, Dunnett's) (Figure 2B). Analysis of the mean time spent in the center of the open field showed a significant main effect for day, F(2, 138) = 7.09, *p* < 0.01, and a significant day × genotype interaction, F(6, 138) = 3.41, *p* < 0.01, while the main effect for genotype was close to significance, F(3, 69) = 2.47, *p* = 0.0692 (Figure 2C). FDD_KI_ mice spent more time in the arena center than WT mice on the first two days (*p*<0.05, Dunnett's). Analysis of the number of entries into the arena center showed significant main effects for day, F(2, 138) = 37.67, *p* < 0.0001, and genotype, F(3, 69) = 4.20, *p* < 0.01, and a near significant interaction between genotype and day, F(6, 138) = 2.04, *p* = 0.0650. *Post-hoc* comparisons (Dunnett's) revealed that FDD_KI_ mice entered the arena center significantly more than did WT mice on the first (*p*<0.001) and second (*p*<0.05) days (Figure 2D). Overall these data indicate that FDD_KI_ mice were generally more active and less anxious than WT, *Aph1BC^−/−^* and FDD_KI_*/Aph1BC^−/−^* mice, while no differences were detected between WT, *Aph1BC^−/−^* and FDD_KI_*/Aph1BC^−/−^* animals.

**Figure 3:**
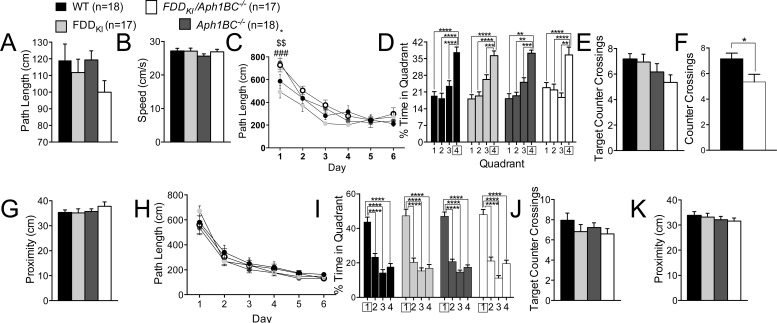
The Morris Water Maze task shows a mild deficit in accuracy of spatial long-term memory 4 month-old FDD_KI_/*Aph1BC*^−/−^ mice Data are expressed as means ± S.E.M. **A.-B.** Performance on the visible platform task. The visible platform task indicates that there were no significant differences among the genotypes in path length traveled (A) or swim speed (B). **C.** Acquisition of spatial reference memory in the hidden platform task. Mean path lengths across 6 daily trials are shown. Significant differences among the genotypes were found only on the first day: * *p* < 0.05, ** *p* < 0.01, *** *p* < 0.001. **D.-F.** Performance on the 60-s probe trial given 2 days after the last acquisition session. (D-F) Performance on the 60-s probe trial given 2 days after the last acquisition session. (D) Percentage of time spent in the four quadrants. ** *p* < 0.01, *** *p* < 0.001, **** *p* < 0.0001 (E) Number of counter crossings in the target quadrant (Quadrant 4). * *p* < 0.05 (F) A separate unpaired t-test showed a significant difference between WT and FDD_KI_*/Aph1BC^−/−^* mice (* *p* = 0.0177). **G.** Average proximity to the former platform location. **H.** Performance in the reversal learning task with a new platform location. Mean path length across 6 daily trials are shown. **I.-K.** Performance on the 60-s probe trail given 2 days after the last reversal learning session. (I) Percentage of time spent in the four quadrants. **** *p* < 0.0001 (J) Number of counter crossings in the target quadrant (Quadrant 1). (K) Average proximity to the former platform location.

Following the open field test, mice were tested in the MWM for spatial reference memory. One WT and one FDD_KI_ mouse were dropped from the experiments permanently due to severe bite wounds and persistent tracking errors caused by its light fur color, respectively. In addition, one FDD_KI_*/Aph1BC^−/−^* mouse died before the task was completed. As shown in Figure 3A and 3B, the visible platform task conducted prior to the reference memory task revealed no significant differences among the four genotype groups in path length traveled, F(3, 66) = 1.43, *p* = 0.2427, or swim speed, F(3, 66) = 0.60, *p* = 0.6177, indicating that none of the mutant mice had any visual or motor deficits relative to WT control at this age. Figure 3C depicts the mean path length traveled by animals during the acquisition phase of the hidden platform task. Two-way ANOVA revealed a significant main effect for day on path length, F(5, 330) = 68.20, *p* < 0.0001, indicating animals' acquisition of reference memory for the platform location. There was also a significant interaction between day and genotype, F(15, 330) = 2.18, *p* < 0.01, while no significant main effect for genotype was found, F(3, 66) = 2.49, *p* = 0.0676. Tukey's comparisons revealed some differences among the genotypes on the first day, with *Aph1BC^−/−^* mice traveling a significantly larger distance than WT mice (*p*<0.05) and FDD_KI_ mice (*p*<0.001), and with FDD_KI_*/Aph1BC^−/−^* mice than FDD_KI_ mice (*p*<0.01). On the probe trial conducted two days after the last acquisition session, the analysis of the percentage of time spent in the four quadrants revealed a significant main effect for quadrant, F(3, 198) = 50.71, *p* < 0.0001, but no significant main effect for genotype, F(3, 66) = 0.55, *p* = 0.6469, or significant quadrant × genotype interaction, F(9, 198) = 1.15, *p* = 0.3293 (Figure 3D). While the percentage of time spent in the target quadrant is the most popular measure of probe trial performance [54], counting the number of times the animal crosses a small area surrounding the former platform position (counter crossings) provides more information on the spatial accuracy with which the exact location of the platform has been encoded [55]. One-way ANOVA did not reveal a significant effect of genotype on the number of counter crossings in the target quadrant, F(3, 66) = 2.00, *p* = 0.1233 (Figure 3E). However, a separate unpaired t-test showed a significant difference between WT and FDD_KI_*/Aph1BC^−/−^* mice (*p* = 0.0177, Figure 3F). There were no significant differences in the average proximity to the original platform location among the genotype groups, F(3, 66) = 0.75, *p* = 0.5282 (Figure 3G). Thus, at 4 months of age, when present together the FDD mutation and the *Aph1BC* deficiency mildly interfered with spatial long-term memory by compromising its accuracy.

In the reversal learning task, in which the location of the hidden platform was moved to the quadrant opposite to the original target quadrant, all the genotypes performed the task in a similar manner during the acquisition phase (Figure 3H), with ANOVA showing a significant main effect for day, F(5, 330) = 102.5, *p* < 0.0001, but no significant main effect for genotype, F(3, 66) = 0.70, *p* = 0.5577, or day × genotype interaction, F(15, 330) = 0.71, *p* = 0.7770. On the probe trial given two days after the last reversal learning session, no differences were found among the genotypes in the percentage of time spent in the quadrants (Figure 3I), with a significant quadrant main effect, F(3, 198) = 140.2, *p* < 0.0001, but no significant main effect for genotype, F(3, 66) = 0.38, *p* = 0.7681, or quadrant × genotype interaction, F(9, 198) = 0.55, *p* = 0.8376. Similarly, no differences were found among the genotypes in the number of counter crossings in the target quadrant (Figure 3J), F(3, 66) = 0.95, *p* = 0.4230, or the average proximity to the target (Figure 3K), F(3, 66) = 0.47, *p* = 0.7029.

**Figure 4:**
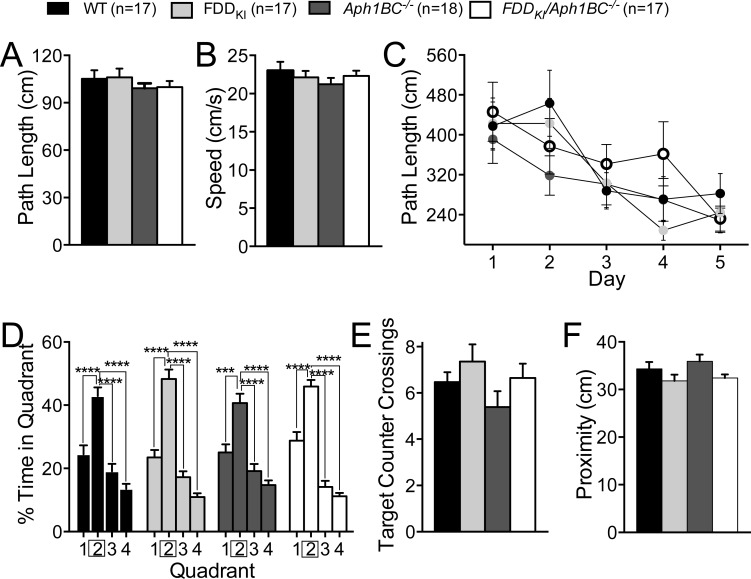
Normal spatial memory in 7-8 month-old mice Data are expressed as means ± S.E.M. **A.-B.** Performance on the visible platform task. There were no significant differences among the genotypes in path length traveled (A) or swim speed (B). **C.** Acquisition of spatial reference memory in the hidden platform task. Mean path lengths across 3 daily trials are shown. No significant differences were found among the genotypes. **D.-F.** Performance on the 60-s probe trial given 2 days after acquisition. (D) Percentage of time spent in the four quadrants. *** *p* < 0.001, **** *p* < 0.0001. (E) Number of counter crossings in the target quadrant (Quadrant 2). (F) Average proximity to the former platform location. No significant differences were found among the genotypes in any of the measures.

The same cohorts of mice were again assessed for spatial reference memory in the Morris water maze at 7-8 months of age. To determine whether genetic manipulations affect learning and memory in an aging dependent manner, it is customary to use different cohorts of animal to perform a task (such as the Morris water maze) at different ages. In this manner, the results are not influenced by learning and/or habituation to the tasks that may occur when the same cohort of mice are subjected to the same task more than once. However, a longitudinal analysis is ethically and economically preferable since it reduces the number of experimental animals. In addition, it eliminates possible confounding effects due to genetic variability between different cohorts of mice. Finally, testing if mice forget, during aging, tasks learned as young adults, mirrors what happens in AD patients. In this new Morris water maze test, the number of daily trials was reduced to three to alter the difficulty of the task. One WT mouse was dropped since it had become too weak to swim. The visible platform task revealed no significant differences among the genotypes in path length traveled, F(3, 65) = 1.36, *p* = 0.2638 (Figure 4A), or swim speed, F(3, 65) = 0.27, *p* = 0.8488 (Figure 4B). In the reference memory task, as shown in Figure 4C, no significant differences among the genotypes were found during acquisition, with two-way ANOVA showing a significant main effect for day, F(4, 260) = 12.41, *p* < 0.0001, but no significant main effect for genotype, F(3, 65) = 0.81, *p* = 0.4932, or day × genotype interaction, F(12, 260) = 0.95, *p* = 0.4957. The probe trial conducted two days after the last acquisition session did not reveal any differences among the genotypes in the percentage of time spent in the four quadrants (Figure 4D), with a significant quadrant main effect, F(3, 195) = 115.4, *p* < 0.0001, but no significant main effect for genotype, F(3, 65) = 1.18, *p* = 0.3257, or quadrant × genotype interaction, F(9, 195) = 1.25, *p* = 0.2670. There was no significant effect of genotype on the number of counter crossings in the target quadrant, F(3, 65) = 1.67, *p* = 0.1828 (Figure 4E), or the average proximity to the target, F(3, 65) = 2.18, *p* = 0.0990 (Figure 4F). Thus, no significant differences were found among the genotypes in the acquisition or retention of spatial reference memory at 7-8 months of age, and the small deficits in counter crossing observed in 4 month-old FDD_KI_*/Aph1BC^−/−^* mice was not seen again at 7-8 months of age, possibly due to learning/habituation to the task.

**Figure 5:**
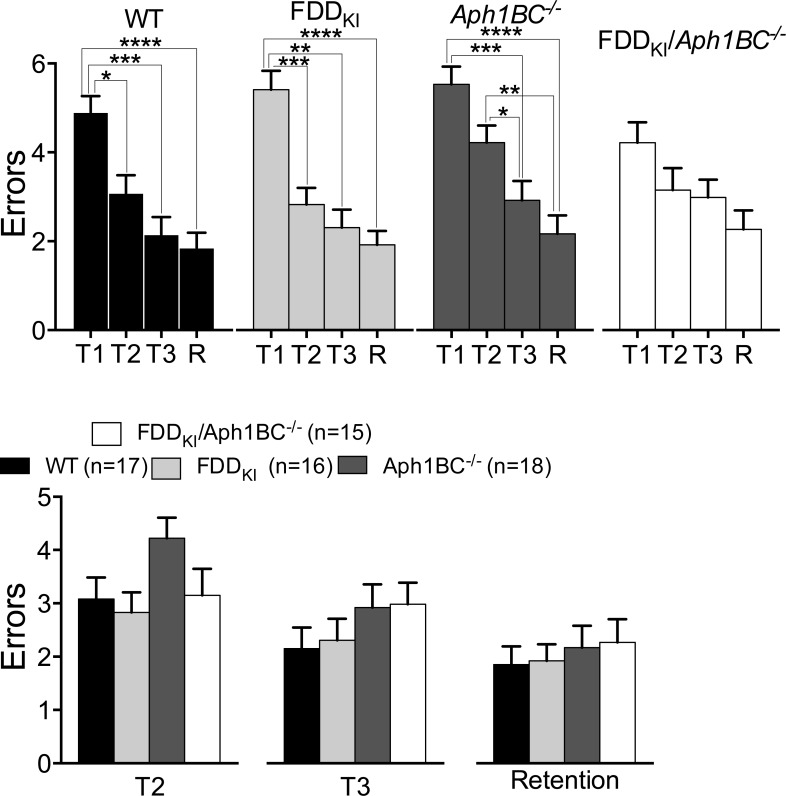
The 8-arm Radial Arm Water Maze task detects possible working memory impairment in FDD_KI_/*Aph1BC*^−/−^ mice at 7-8 months of age Data are expressed as means ± S.E.M. **A.** Number of errors on 3 daily acquisition trials averaged across the last 3 test days. The performance by WT, FDD_KI_, and *Aph1BC^−/−^* mice improved across trials to a greater degree, compared to FDD_KI_*/Aph1BC^−/−^* mice. *p < 0.05, **p < 0.01, ***p < 0.001, **** *p* < 0.0001. **B.** Number of errors on trials 2, 3 and retention (R) given 30 min after the third acquisition trial. No significant differences were found among the genotypes.

Following the completion of the probe trial at 7-8 months, mice were subjected to a working memory task in the eight-arm radial arm water maze. Mice were initially given four consecutive daily acquisition trials with a 15 seconds inter-trial interval. However, it became apparent after six days of training with this procedure that most mice lacked the physical strength required for maintaining their performance in the maze during the four consecutive acquisition trials, and that their performance was not improving after six days (data not shown). Accordingly, the procedure was modified such that mice would be given a 6-min inter-trial interval in the holding cage, and that three, instead of four, acquisition trials would be given before the retention trial. After a break on the seventh day, mice were tested for five more days with this new procedure and the results pertaining to these last five days are shown in Figure 5. Repeated measures one-way ANOVA (RM one-way ANOVA) found a significant main effect for trial in WT [F (2.065, 33.04) = 18.37, *p* < 0.0001)], FDD_KI_ [F (2.320, 37.12) = 19.88, *p* < 0.0001)] and *Aph1BC^−/−^* [F (2.312, 39.31) = 22.14, *p* < 0.0001)], but not in FDD_KI_*/Aph1BC^−/−^* mice [F (2.033, 28.46) = 3.218, p=0.0542)]. A *post hoc* multiple comparison of the mean of each trial to the mean of every other trial (Tukey's multiple comparisons test) indicated that performance by WT and FDD_KI_ mice improved significantly between Trials 1 and 2 (*p* < 0.05 for WT and *p* < 0.01 for FDD_KI_), Trials 1 and 3 (*p* < 0.001 for WT and *p* < 0.01 for FDD_KI_) and Trials 1 and R (*p* < 0.0001 for both genotypes). The performance of *Aph1BC^−/−^* mice improved significantly between Trials 1 and 3, 1 and R (*p* < 0.0001) as well as 2 and 3 (*p* < 0.05),and 2 and R (*p* < 0.01). On the other hand, this *post hoc* multiple comparison analysis was not performed for the FDD_KI_*/Aph1BC^−/−^* mice since the RM one-way ANOVA analysis did not find a significant main effect for trial in these mice. Overall, these observations indicated that the magnitude of improvement in performance was the smallest in FDD_KI_/*Aph1BC^−/−^* mice, compared to WT, FDD_KI_, or *Aph1BC^−/−^* mice. This difference may reflect working memory impairment in FDD_KI_*/Aph1BC^−/−^* mice, or might have resulted from the fact that FDD_KI_/*Aph1BC^−/−^* mice actually made fewer errors than mice of the other genotypes on the first trial, thereby making the gain smaller.

### The FDD and *Aph1BC^−/−^* mutations induce mild deficits in spatial long-term memory in middle age

**Figure 6:**
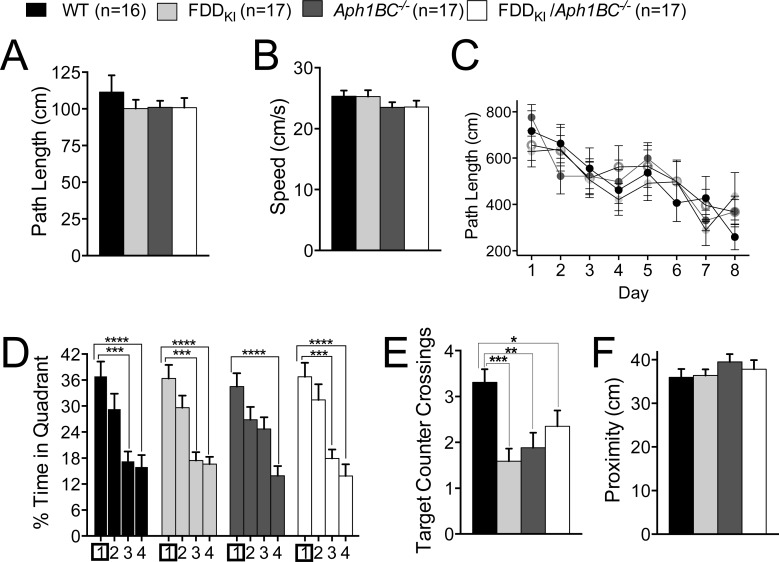
Mild deficit in accuracy of spatial long-term memory in 15 month-old FDD_KI_, *Aph1BC*^−/−^ and FDD_KI_/*Aph1BC*^−/−^ mice Data are expressed as means ± S.E.M. **A.-B.** Performance on the visible platform task. There were no significant differences among the genotypes in path length traveled (A) or swim speed (B). **C.** Acquisition of spatial reference memory in the hidden platform task. Mean path lengths across 2 daily trials are shown. No significant differences were found among the genotypes. **D.-F.** Performance on the 30-s probe trial given 2 days after acquisition. **D.** Percentage of time spent in the four quadrants. *** *p* < 0.001, **** *p* < 0.0001. **E.** Number of counter crossings in the target quadrant (Quadrant 1). WT mice crossed the target counter significantly more than FDD_KI_ or *Aph1BC^−/−^* mice. *** *p* < 0.01, WT *vs*. FDD_KI_, ** *p* < 0.01, WT *vs*. *Aph1BC^−/−^*; * *p* < 0.05, WT *vs*. FDD_KI_/*Aph1BC^−/−^*. **F.** Average proximity to the former platform location.

At 12 months of age, another reference memory task was given. For this task, the number of daily trials was further reduced to two per day, and the length of the probe trial was reduced to 30 s. In addition, the visible platform task was given after the completion of the probe trials. One WT mouse that displayed extensive and persistent thigmotaxis during the reference memory task was excluded from the data analysis. Also, one *Aph1BC^−/−^* mouse was eliminated because it had developed rectal prolapse. Again, the visible platform task did not find any differences among the genotypes in path length traveled, F(3, 63) = 0.81, *p* = 0.4911 (Figure 6A), or swim speed, F(3, 63) = 0.81, *p* = 0.4908 (Figure 6B). In the reference memory task, mice of all the genotype learned the location of the hidden platform in a similar manner (Figure 6C). ANOVA found a significant main effect for day, F(7, 441) = 11.84, *p* < 0.0001, but no significant genotype main effect, F(3, 63) = 0.10, *p* = 0.9576, or day × genotype interaction, F(21, 441) = 0.75, *p* = 0.7773. On the probe trial given two days after the last acquisition session, no significant differences were found among the genotypes in the analysis of the percentage of time spent in the quadrants (Figure 6D), with a significant main effect for quadrant, F(3, 189) = 35.30, *p* < 0.0001, but no significant genotype main effect, F(3, 63) = 1.21, *p* = 0.3123, or quadrant × genotype interaction, F(9, 189) = 0.65, *p* = 0.7571. However, the analysis of the number of counter crossings in the target quadrant revealed a significant effect of genotype, F(3, 63) = 5.80, *p* < 0.01 (Figure 6E). *Post-hoc* comparisons (uncorrected Fisher's LSD) revealed that WT mice crossed the counter in the target quadrant significantly more often than FDD_KI_ mice (*p* < 0.001), *Aph1BC^−/−^* (*p* < 0.01) and FDD_KI_*/Aph1BC^−/−^* mice (*p* < 0.05). There was no significant effect of genotype on the average proximity to the former platform location, F(3, 63) = 0.79, *p* = 0.5065 (Figure 6F). Thus, at 12 months of age, the FDD mutation and the *Aph1BC* deficiency both mildly interfered with spatial long-term memory by compromising its accuracy.

**Figure 7:**
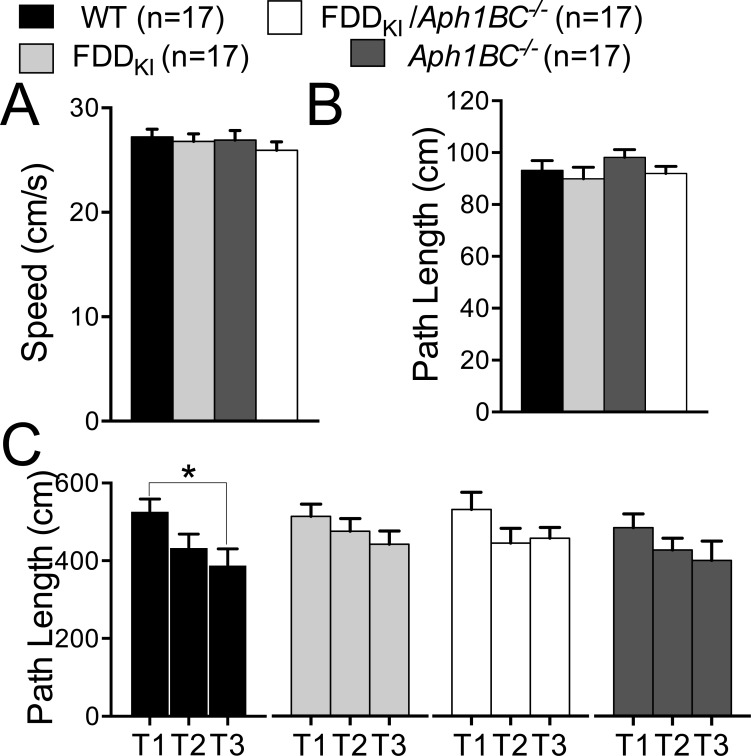
Mild deficit in working memory in FDD_KI_, *Aph1BC*^−/−^ and FDD_KI_/*Aph1BC*^−/−^ mice at 15 months of age Data are expressed as means ± S.E.M. **A.-B.** Performance in the visible platform task. There were no significant differences among the genotypes in path length traveled (A) or swim speed (B). **C.** Path length traveled on the 3 daily trials of the working memory task averaged across the last 3 test days. WT mice significantly reduced the path length traveled to reach the platform between trial 1 and 3 (* *p* < 0.05), while mice of the other 3 genotypes did not.

Mice were tested for spatial working memory in Morris water maze at 15 months of age. As shown in Figure 7A and 7B, the visible platform task given at this age found no significant effect of genotype on path length traveled, F(3, 64) = 0.37, *p* = 0.7743, or swim speed, F(3, 64) = 0.66, *p* = 0.5797. In the working memory task, in which the location of the hidden platform was changed daily, we measured the path-length to target (Figure 7C). RM one-way ANOVA found a significant main effect for trial in WT [F (1.984, 29.76) = 5.004, *p* = 0.0136)], but not in FDD_KI_ [F (1.948, 31.17) = 1.822, *p* = 0.1793)], *Aph1BC^−/−^* [F (1.943, 31.09) = 2.749, *p* = 0.0809] and FDD_KI_*/Aph1BC^−/−^* mice [F (1.655, 26.48) = 2.180, *p=*0.14)]. A *post hoc* multiple comparison of the mean of each trial to the mean of every other trial (Tukey's multiple comparisons test) indicated that only the performance of WT mice improved significantly between Trials 1 and 3 (*p* < 0.05). This test suggests that, at 15 months of age, the FDD mutation and the *Aph1BC* deficiency both mildly interfered with working memory.

### Learning and memory deficits are caused by the Aph1BC deficiency and the FDD mutation at old age (18-19 months)

**Figure 8:**
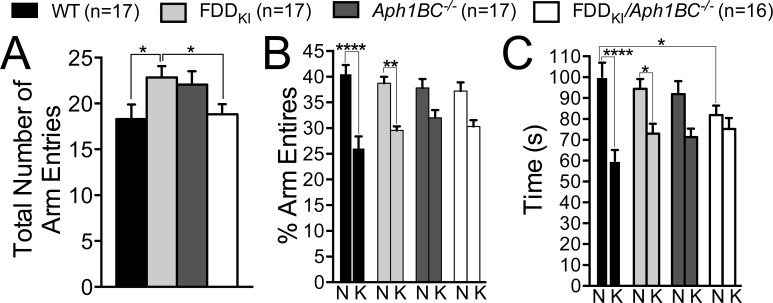
The two-trial Y-maze test showed mild deficit of short-term spatial recognition memory in FDD_KI_, *Aph1BC*^−/−^ and FDD_KI_/*Aph1BC*^−/−^ mice at 18-19 months of age Data are expressed as means ± S.E.M. **A.** Total number of arm entries. FDD_KI_ mice made significantly more arm entries than WT or FDD_K*I*_*/Aph1BC^−/−^* mice (*p < 0.05). **B.** Percentage of entries into the novel (N) and known (K) arms. WT and, to a smaller degree, FDD_KI_ mice entered the novel arm significantly more than the known arm, while FDD_KI_*/Aph1BC^−/−^* or *Aph1BC^−/−^* mice did not (**p < 0.01; **** *p* < 0.0001). **C.** Time spent in the novel and known arms. WT mice and, to a smaller degree, FDD_KI_ mice spent significantly more time in the novel arm than in the known arm, while FDD_KI_*/Aph1BC^−/−^* or *Aph1BC^−/−^* mice did not (*p < 0.05; **** *p* < 0.0001). In addition, there was a significant difference in time spent in the novel arm between WT and FDD_KI_/*Aph1BC^−/−^* (*p < 0.05).

At 18-19 months of age, mice were given a series of tests. The first test, conducted at 18-19 months of age, evaluated mice in the two-trial Y-maze test task for spatial recognition memory. One FDD_KI_/*Aph1BC^−/−^* mouse had died before reaching this age. Figure 8A depicts the mean number of arm entries during the 5-min test trial, which is an index for animals' total activity levels. ANOVA found a small but significant effect of genotype, F(3, 63) = 2.77, *p* =0.0490 < 0.05, and Fisher's LSD comparison test showed that *FDD_KI_* mice were more mobile than WT and *FDD_KI_/Aph1BC^−/−^* mice (*p* < 0.05). Fisher's test was used here because, despite the significant overall genotype effect, no significant differences among the genotypes were detected by corrected comparison tests. Given the significant genotype effect on the number of total arm entries, the percentage of entries into each arm, instead of raw numbers of arm entries, was used to analyze animals' preference for the novel arm vs. the known arm. As shown in Figure 8B, the analysis of the percentage of entries into the novel and known arms found a significant main effect for arm, F(1, 63) = 43.34, *p* < 0.0001, but no significant genotype main effect, F(3, 63) = 0.78, *p* =0.5076, or arm × genotype interaction, F(3, 63) = 1.96, *p* =0.1295. Sidak's multiple comparisons between the arms revealed that the novel arm was entered significantly more than the known arm by WT mice and FDD_KI_ mice, but not by FDD_KI_*/Aph1BC^−/−^* mice or *Aph1BC^−/−^* mice, and that the level of statistical significance was much higher for WT mice (*p* < 0.0001) than for FDD_KI_ mice (*p* < 0.01). As can be seen in Figure 8C, the analysis of the time spent in the novel and known arms found a significant main effect for arm, F(1, 63) = 29.63, *p* < 0.0001, as well as a significant interaction between arm and genotype, F(3, 63) = 2.82, *p* < 0.05, while showing no significant main effect for genotype, F(3, 63) = 0.43, *p* = 0.7345. *Post-hoc* comparisons across the genotypes (Dunnett's) revealed that WT mice spent significantly more time in the novel arm than did FDD_KI_*/Aph1BC^−/−^* mice (*p* < 0.05). In addition, comparisons between the arms (Sidak's) showed that WT mice spent significantly more time in the novel arm than in the known arm (*p* < 0.0001). FDD_KI_ mice also spent significantly more time in the novel arm than in the known arm, but to a much smaller degree (*p* < 0.05) than WT mice. By contrast, the difference in time spent between the novel and known arms were not statistically significant for FDD_KI_*/Aph1BC^−/−^* or *Aph1BC^−/−^* mice. In sum, the results of the two-trial Y-maze task demonstrated that WT mice and, to a lesser extent, FDD_KI_ mice distinguished between the novel and familiar arms better than FDD_KI_*/Aph1BC^−/−^* or *Aph1BC^−/−^* mice after a 1-h retention interval, indicating that the *Aph1BC* deficiency, and to a lesser extent the FDD mutation, leads to impairment of short-term spatial recognition memory.

**Figure 9:**
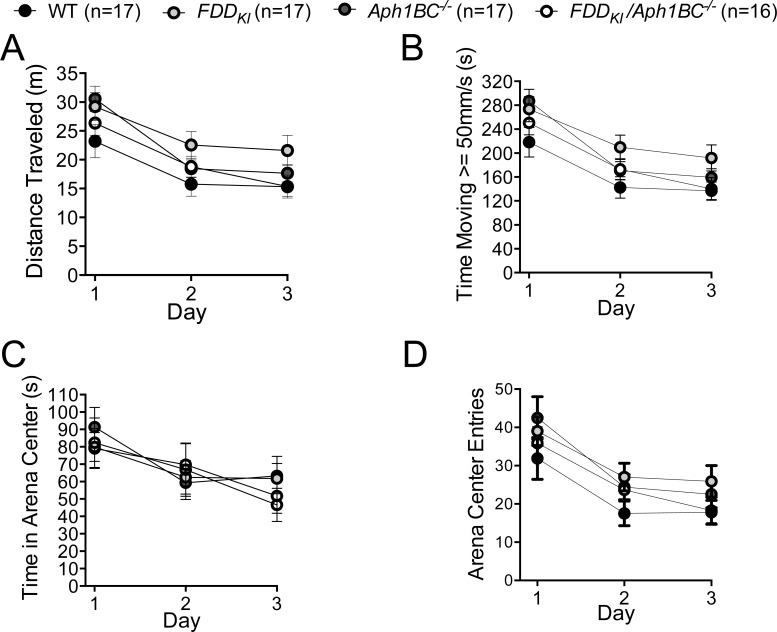
Open Field test on mice at 18-19 months of age Data are expressed as means (± S.E.M.) during the 10-min testing period over 3 days. **A.** Total distance traveled. **B.** Amount of time in which the animal ambulated at speed greater than 50 mm/s. **C.** Amount of time the animal spent in the center of the arena (20 cm × 20 cm). **D.** Total number of entries into the arena center. No significant differences were found among the genotypes in any of the measures.

Next we analyzed the general locomotor activity levels in the open field at 18-19 months. There was a main effect for day in the total distance traveled, F(2, 126) = 85.01, *p* < 0.0001, but no significant main effect for genotype, F(3, 63) = 2.03, *p* = 0.1186, or interaction between day and genotype, F(6, 126) = 1.83, *p* = 0.0977 (Figure 9A). There was a main effect for day in the amount of time in which the animal ambulated at speed greater than 50 mm/s, F(2, 126) = 94.25, *p* < 0.0001, but no significant genotype main effect, F(3, 63) = 2.24, *p* = 0.0919, or day × genotype interaction, F(6, 126) = 1.62, *p* = 0.1466 (Figure 9B). We detected a significant main effect for day, F(2, 126) = 10.66, *p* < 0.0001, but no significant main effect for genotype, F(3, 63) = 0.09, *p* = 0.9667, or day × genotype interaction, F(6, 126) = 0.66, *p* = 0.6770 in the mean time spent in the center of the arena (Figure 9C). The total number of entries into the arena center showed significant main effect for day, F(2, 126) = 35.27, *p* < 0.0001, but no significant genotype main effect, F(3, 63) = 1.51, *p* = 0.2212, or day × genotype interaction, F(6, 126) = 0.45, *p* = 0.8419 (Figure 9D).

**Figure 10:**
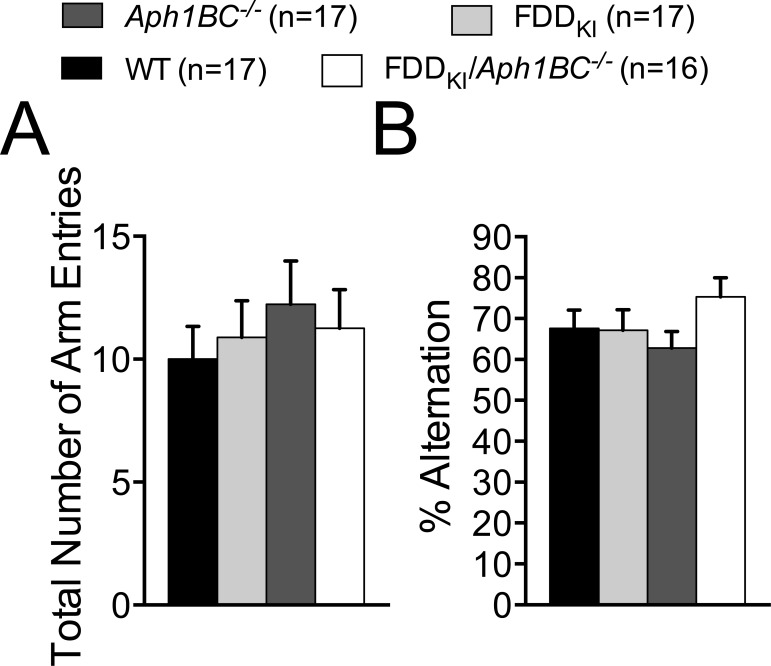
Y-maze spontaneous alternation test at 18-19 months of age Data are expressed as means ± S.E.M. **A.** Total number of arm entries. **B.** Percentage of alternations. Six mice with less than three arm entries are not included in (B). No significant differences were found among the genotypes in either measure.

Following the open field test, the spontaneous alternation test was conducted in the Y-maze to assess animals' spatial working memory. Analysis of the total number of arm entries during the 5-min testing period showed no significant effect of genotype was detected, F(3, 62) = 0.36, *p* = 0.7811 (Figure 10A). Since the minimum number of arm entries needed for a complete alternation is three, six mice that had less than three arm entries in total (2 WT, 1 FDD_KI_, 1 FDD_KI_/*Aph1BC^−/−^*, and 2 *Aph1BC^−/−^*) were not included in the analysis of the percentage of alternations, which showed no significant effect of genotype, F(3, 57) = 1.26, *p* = 0.2969 (Figure 10B).

**Figure 11:**
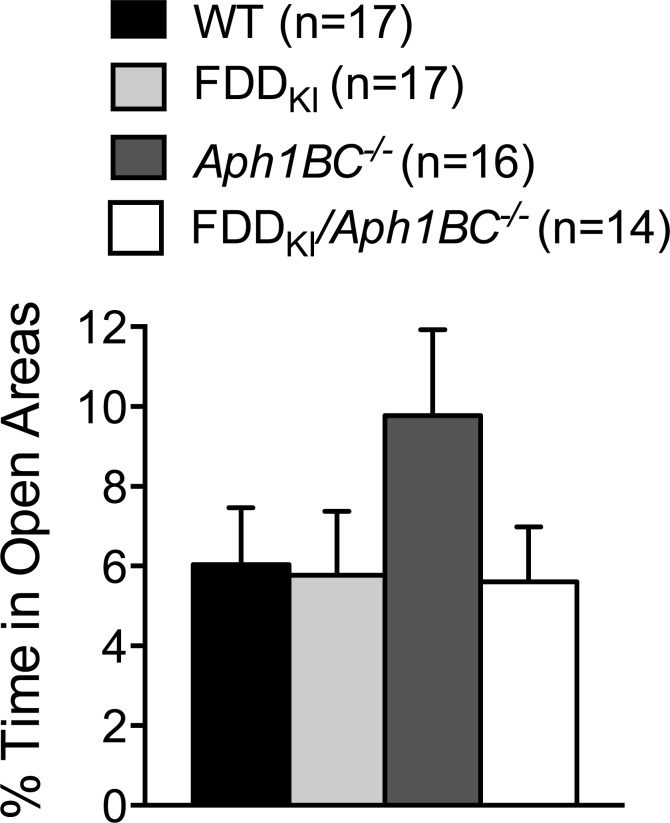
Elevated Zero maze test on mice at 18/-9 months of age Mean (± S.E.M.) percentage of time spent in the open areas of the elevated zero maze. There were no significant differences among the genotypes.

Next, we used the elevated zero maze to test for anxiety-like behavior. As shown in Figure 11, while *Aph1BC^−/−^* mice spent more time in open areas than mice of the other genotypes on average, the genotype effect did not reach statistical significance, F(3, 60) = 1.396, *p* = 0.2527. Three animals (2 FDD_KI_/*Aph1BC^−/−^* and 1 *Aph1BC^−/−^*) fell off the open areas of the maze during testing and were excluded from the data analysis.

**Figure 12:**
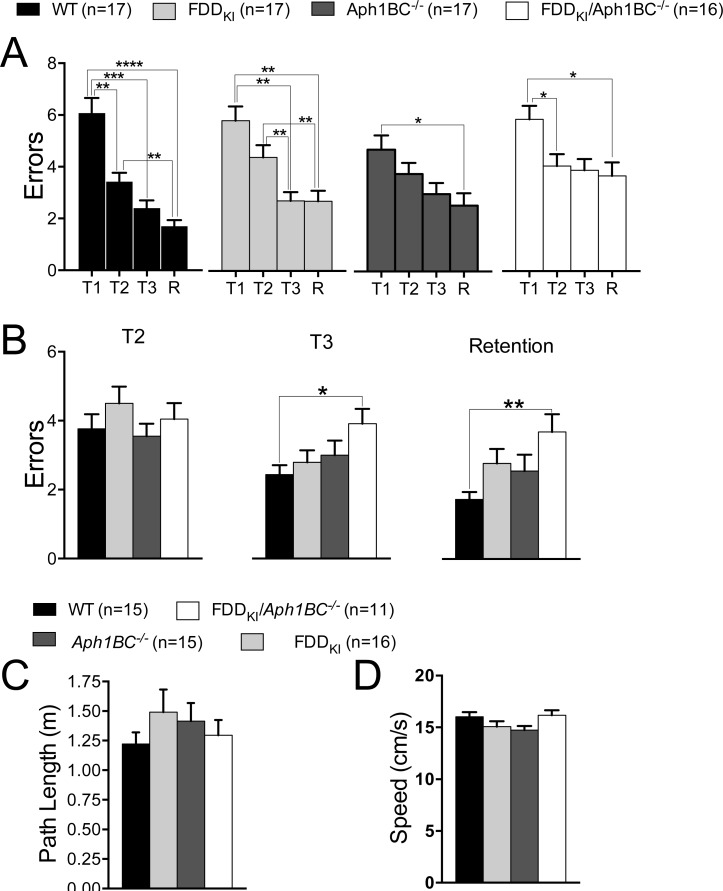
Mild spatial working memory deficits in 18-19 month-old FDD_KI_, *Aph1BC*^−/−^ and FDD_KI_/*Aph1BC*^−/−^ mice Data are expressed as means ± S.E.M. (A) Number of errors on 3 daily acquisition trials averaged across days 6-9 of testing. (B) Number of errors on trials 2, 3 and retention (R) given 30 min after the third acquisition trial. The visible platform task showed no significant differences among the genotypes in path length traveled (C) or swim speed (D). *p < 0.05, **p < 0.01, ***p < 0.001, **** *p* < 0.0001.

Mice were then evaluated for spatial working memory in the 6-arm radial arm water maze. RM one-way ANOVA found a significant main effect for trial in WT [F (2.025, 32.40) = 28.12, *p* < 0.0001)], FDD_KI_ [F (2.082, 33.32) = 13.32, *p* < 0.0001)], *Aph1BC^−/−^* [F (2.437, 38.99) = 4.542, *p* = 0.012)] and FDD_KI_*/Aph1BC^−/−^* mice [F (2.738, 41.07) = 4.680, *p=*0.0081)] (Figure 12A). A *post hoc* multiple comparison of the mean of each trial to the mean of every other trial (Tukey's multiple comparisons test) indicated that performance by WT mice improved significantly between Trials 1 and 2 (*p* < 0.01), 1 and 3 (*p* < 0.001), 1 and R (*p* < 0.0001) as well as between Trials 2 and R (*p* < 0.01). The FDD_KI_ mice improved between Trials 1 and 3 and 1 and R as well as between Trials 2 and 3 and 2 and R (*p* < 0.01). As for FDD_KI_/*Aph1BC^−/−^* and *Aph1BC^−/−^* mice, their performance improved significantly between Trials 1 and R (*p* < 0.05). However, it is worth noting that *Aph1BC^−/−^* mice actually made fewer errors than mice of the other genotypes on the first trial, thereby making the gain smaller. On the other hand, the performance by FDD_KI_*/Aph1BC^−/−^* mice significantly improved between Trials 1 and 2,1 and R, but the degree of significance was much smaller (*p* < 0.05) as compared to WT and FDD_KI_ mice.

We also compared the number of errors made by mice of the four genotypes at Trials 2, 3 and R (Figure 12B). Ordinary one-way ANOVA found significant effect of genotype at Trials 3 [F (3, 63) = 2.751, *p* = 0.05)] and R [F (3, 63) = 3.616, *p* = 0.00178)], but not 2 [F (3, 63) = 0.9047, *p* = 0.4439)]. A *post hoc* multiple comparison of the mean of each genotype to the mean of every other genotype (Tukey's multiple comparisons test) indicated that WT mice committed significantly fewer errors than FDD_KI_*/Aph1BC^−/−^* mice both at Trials 3 (*p* < 0.05) and R (*p* < 0.01). The differences between WT *vs.* FDD_KI_, WT *vs. Aph1BC^−/−^*, FDD_KI_*/Aph1BC^−/−^ vs.* FDD_KI_ and FDD_KI_*/Aph1BC^−/−^ vs. Aph1BC^−/−^* were not statistically significant. Altogether, these observations indicated that the degree of improvement in performance across trials during acquisition and retention was the greatest for WT mice, followed by FDD_KI_, *Aph1BC^−/−^* and FDD_KI_/*Aph1BC^−/−^* mice in the order named. These results further indicate that the FDD mutation and deletion of *Aph1BC* induces spatial working memory deficits. These data are also in accordance with the results of a previous study showing that the *Aph1BC^−/−^* mutation induces spatial working memory deficits in mice of the F2 generation of the C57BL/6J–129/Ola hybrids [53].

After the radial arm water maze test, mice were assessed for possible visual or motor deficits in the visible platform task. Ten animals (2 WT, 1 FDD_KI_, 5 FDD_KI_*/Aph1BC^−/−^*, and 2 *Aph1BC^−/−^*) were mistakenly sacrificed prematurely after the radial arm water maze and before the visual platform task. As shown in Figure 12C and 12D, the visible platform task did not reveal any visual or motor deficits at this age. There was no significant effect of genotype on path length traveled, F(3, 53) = 1.13, *p* = 0.3467, or swim speed, F(3, 53) = 2.19, *p* = 0.0999.

**Figure 13:**
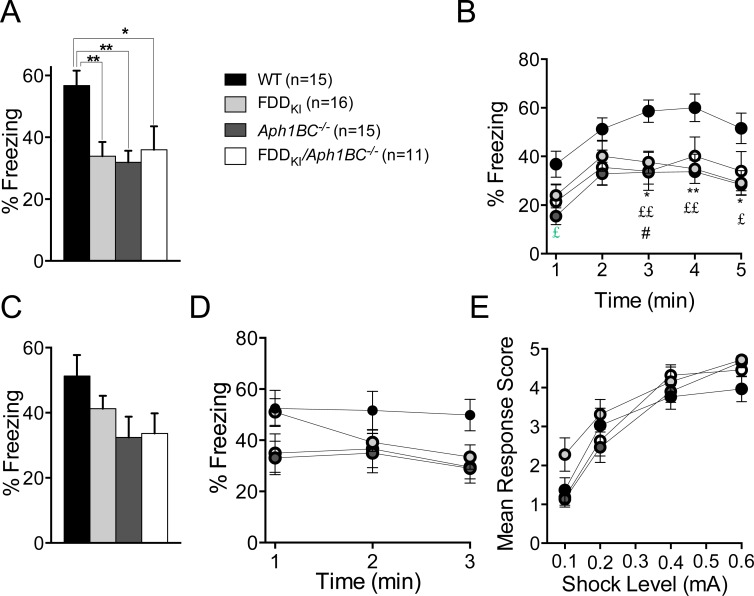
Deficit in long-term contextual fear memory in FDD_KI_, *Aph1BC*^−/−^ and FDD_KI_/*Aph1BC*^−/−^ mice at 18-19 months of age Data are expressed as means ± S.E.M. **A.** Percentage of freezing during the last 3-m period of the contextual test. WT mice froze significantly more than mice of the other genotypes. *p < 0.05, WT *vs*. FDD_KI_/*Aph1BC^−/−^*; ** *p* < 0.01 WT *vs*. FDD_KI_ and WT *vs*. *Aph1BC^−/−^*. **B.** Time course of freezing behavior during the contextual test in 1-m time bins. There was a significant genotype main effect (*p* < 0.01), with WT freezing more than the other genotypes. *p < 0.05, ** *p* < 0.01, WT *vs*. FDD_KI_; £ *p* < 0.05, ££ *p* < 0.01, WT *vs*. *Aph1BC^−/−^*; # *p* < 0.05, WT *vs*. FDD_KI_*/Aph1BC^−/−^*. **C.** Percentage of freezing during the 3-m tone presentation in the altered context in the cued test. WT mice froze more than mice of the other genotypes, although not significantly. **D.** Time course of freezing behavior during tone presentation in the cued test. Only WT mice remained frozen for the entire period. **E.** Sensitivity to varying intensity levels of foot shock. Mice of all the genotypes reacted to shock at the four intensity levels in a similar manner.

Finally, mice were tested for contextual and cued fear memory using the fear conditioning paradigm. As is indicated in Figure 13A and 13B, in the test for contextual fear memory conducted 24 h after conditioning, WT mice exhibited freezing behavior significantly more than mice in the other genotype groups. ANOVA revealed a significant effect of genotype on the mean percentage of freezing during the last 3 min of the test session, F(3, 53) = 5.45, *p* < 0.01, and *post-hoc* comparisons (Tukey's) indicated that WT mice froze significantly more than FDD_KI_ (*p* < 0.01), *Aph1BC^−/−^* (*p* < 0.01), and FDD_KI_*/Aph1BC^−/−^* (*p* < 0.05) mice (Figure 13A). As can be seen in Figure 13B, the analysis of the time course of the percentage of freezing in 1-min bins during the contextual test also showed a significant main effect for genotype, F(3, 53) = 4.75, *p* < 0.01, in addition to a significant main effect for time, F(4, 212) = 20.55, *p* < 0.0001, while finding no significant time × genotype interaction, F(12, 212) = 0.89, *p* = 0.5557. Tukey's multiple comparison test further indicated that WT mice froze significantly more than FDD_KI_ mice in the last three minutes (*p* < 0.01 for the fourth min; *p* < 0.05 for the third and fifth min), more than *Aph1BC^−/−^* mice in the first minute (*p* < 0.05) and the last three minutes (*p* < 0.01 for the third and fourth min; *p* < 0.05 for the fifth min), and more than FDD_KI_*/Aph1BC^−/−^* mice in the third minute (*p* < 0.05). In the test for cued fear memory conducted 24 h after the contextual test, no significant effect of genotype was found on the percentage of freezing during the presentation of the tone CS, F(3, 53) = 2.27, *p* = 0.0910 (Figure 13C). However, as can be seen in Figure 13D, which shows the time course of freezing during tone presentation, while WT mice remained frozen throughout the 3-min presentation of the tone, FDD_KI_ mice, which were initially as frozen as WT mice, became more mobile towards the end of this period, and FDD_KI_*/Aph1BC^−/−^* and *Aph1BC^−/−^* mice froze less than WT mice for the entire period. Yet, the analysis of the time course showed a significant main effect for time, F(2, 106) = 6.97, *p* < 0.01, but no significant main effect for genotype, F(3, 53) = 2.27, *p* = 0.0910, or time × genotype interaction, F(6, 106) = 2.09, *p* = 0.0603. Nevertheless, the time × genotype interaction approached significance, and when one-way ANOVA was performed on the percentage of freezing during the first, second, and third 1-min time periods separately, while no significant genotype effect was detected in the first minute, F(3, 53) = 2.47, *p* = 0.0720, or the second minute, F(3, 53) = 1.34, *p* = 0.2712, there was a significant genotype effect in the third minute, F(3, 53) = 3.25, *p* < 0.05. Dunnett's comparison test further revealed a significant difference between WT and FDD_KI_*/Aph1BC^−/−^* mice (*p* < 0.05), as well as between WT and *Aph1BC^−/−^* mice (*p* < 0.05), in the last minute of tone presentation. Lastly, a day after the completion of the test for cued fear memory, all the experimental mice were evaluated for shock sensitivity. As shown in Figure 13E, mice of all the genotypes reacted to electric shock at the four intensity levels in a similar manner, with ANOVA showing a significant main effect for shock level, F(3, 159) = 97.70, *p* < 0.0001, but no significant main effect for genotype, F(3, 53) = 1.60, *p* = 0.2010, or shock level × genotype interaction, F(159, 159) = 1.45, *p* = 0.1709. While the amygdala is required and sufficient for fear conditioning to discrete sensory cues such as a tone, rapid formation of conditioned fear responses to environmental context is additionally mediated by the hippocampus [56-59]. Thus, our results indicate that both the FDD and *Aph1BC^−/−^* mutations interfere with mnemonic information processing at the level of the hippocampus for the formation of long-term contextual fear memory at this age, while the *Aph1BC^−/−^* mutation additionally has adverse effects on fear memory formation in the amygdala.

## DISCUSSION

The clinical symptoms of AD include progressive loss of memory, thinking and language skills, as well as other behavioral changes. Short-term memory is the first to fail in AD patients. These clinical symptoms are accompanied by neuronal degeneration. Although it is widely believed that the disease is precipitated by the insurgence of brain lesions, such as Aβ amyloid plaques and neurofibrillary tau tangles, whether these alterations of tau and Aβ metabolism cause the debilitating clinical symptoms of AD and FDD is still unclear.

Because of the uncertainty concerning the pathogenic biochemical mechanisms of AD and related dementias, and considering that to improve the quality of life of AD patients -and caregivers- we need to either reverse or slow down the progression of the clinical symptomatology, we focused our analysis on learning and memory in our animal model of disease. In this study, the same cohort of mice of WT, FDD_KI_, FDD_KI_/*Aph1BC^−/−^*, and *Aph1BC^−/−^* mice was assessed longitudinally for possible deficits in learning and memory from ~4 to ~19 months of age. FDD_KI_/*Aph1BC^−/−^* mice presented mildly compromise accuracy of spatial long-term memory and working short-term memory impairments at 4 and 8 months of age, respectively. Deficits in spatial and working memory were, at later ages, observed also in single mutant FDD_KI_ and *Aph1BC^−/−^* mice. Analysis of older animals showed that spatial recognition memory and long-term contextual fear memory were impaired in FDD_KI_, FDD_KI_/*Aph1BC^−/−^*, and *Aph1BC^−/−^* mice. Overall, this study shows that; 1) the *Aph1BC^−/−^* mutation does not rescue memory deficits in *FDD_KI_* mice, as would have been expected if Aβ played a pathogenic role in *FDD_KI_* mice; 2) in contrast, the FDD mutation and the *Aph1BC^−/−^* deletion cause similar behavioral deficits in spatial memory and appear to have a small additive negative effect, at least on spatial long-term memory and working short-term memory, that is more pronounced at younger ages. Performing the two-trial Y-maze and the fear conditioning tasks in younger mice could unveil whether the FDD mutation and the *Aph1BC* deletion have also a negative additive effect on spatial recognition memory and long-term contextual fear memory. These observations are consistent with a pathological role of β-CTF, which is augmented in FDD_KI_ mice due to increased production and in *Aph1BC^−/−^* mice due to reduced turnover.

We have previously shown that inhibition of β-processing of APP rescues memory and synaptic impairments of FDD_KI_ mice acutely and transiently, suggesting that increased β-cleavage of APP, perhaps in the synaptic cleft, during synaptic events leading to LTP and memory acquisition, leads to memory/synaptic deficits in FDD_KI_ mice [25, 26]. This evidence, together with the data showing memory deficits in *Aph1BC^−/−^* mice, suggest that either increasing the rate of production (like in FDD_KI_ mice) or decreasing the rate of clearance (as in *Aph1BC^−/−^* mice) of β-CTF during synaptic transmission might lead to cognitive impairments. Future experiments will have to test whether these hypotheses are correct and whether the additive adverse effects on spatial long-term memory in young mice of the FDD_KI_ and the *Aph1BC^−/−^* mutations are due to a synergistic effect on β-CTF levels transiently produced during synaptic transmission.

FDD_KI_ and *Aph1BC^−/−^* mice show a few distinct phenotypes. In fear conditioning tests, mice carrying the *Aph1BC* deletion (FDD_KI_*/Aph1BC^−/−^* and *Aph1BC^−/−^* mice) showed a mild impairment of cued fear memory, a task completely dependent on the functional integrity of the amygdala, as compared to FDD_KI_ and WT animals. This result is not surprising since several differences exist between FDD_KI_ and *Aph1BC^−/−^* mice. First, the Aph1B/C deficiency causes a reduced clearance of β-CTF that leads to accumulation of β-CTF and a concomitant reduction in the β-CTF metabolites AID/AICD and Aβ. On the contrary, in FDD_KI_ mice the loss of mBRI2 leads to an increased production of all these APP metabolites. Second, in *Aph1BC^−/−^* mice processing of another γ-secretase substrate, Neuregulin-1 [53] is reduced. Thus, behavioral deficits caused by the deletion of *Aph1B* and *Aph1C*, but not by the FDD mutation, could be attributed to reduction in AID/Aβ and/or reduction of processing of Neuregulin-1.

## MATERIALS AND METHODS

### Subjects

All behavioral experiments were conducted by using male littermates of the F2 generation of the C57BL/6J–129 hybrid mice as subjects. Mice were generated and maintained at the Animal facility of the Albert Einstein College of Medicine. Four genotypes, *Aph1BC^−/−^*, *FDD_KI_*, *FDD_KI_/Aph1BC^−/−^*, and wild type (WT), of F2 mice (n=11-19 per genotype) were evaluated for behavior. *Aph1BC^−/−^* mice have been previously described [51]. *FDD_KI_* mice carried one mutant and one wild type *BRI2/ITM2b* allele [19]. Upon weaning, all mice were implanted with electronic chips (PharmaSeq, Monmouth Junction, NJ) subcutaneously on the tail for identification purposes, and their identity was regularly checked during testing periods. Animals were group-housed in plastic cages with *ad libitum* access to food and water in a temperature- and humidity-controlled animal care facility with a 12-h light/12-h dark cycle. All experimental procedures were in accordance with the National Institutes of Health guidelines and approved by the Institutional Animal Care and Use Committee (IACUC) at the Albert Einstein College of Medicine in animal protocol number 20130509.

### Behavioral experimental procedures

All mice were extensively handled prior to the start of behavioral testing. All behavioral testing was conducted during the light cycle. On each testing day, animals were transported to a behavioral testing suite in their home cages and allowed to acclimate for at least 30 min prior to the start of testing. The experimenter was not blind to the genotypes of the animals in tests conducted at 4, 7-8, 12 and 15 months of age but was made blind in tests conducted at 18-19 months. All measurements were taken automatically by video tracking software.

### Elevated zero maze

Mice were assessed for anxiety-like behavior on the elevated zero maze initially at 4 months of age and again at 18-19 months. The zero maze (Stoelting, Wood Dale, IL) consisted of an annular platform (inner diameter 50 cm, width 5 cm) elevated to 50 cm above the ground level, divided equally into four quadrants. Two opposite quadrants were enclosed by walls (15 cm high) on both the inner and outer edges of the platform (closed areas), while the remaining two opposite quadrants were open without walls (open areas). Light levels over the maze were kept constant at approximately 50 lx in the open areas and 30 lx in the closed areas. Mice were placed individually in a closed quadrant and allowed to explore the maze freely for 5 min. The behavior of mice was monitored using a video camera, and their movements were analyzed with a video tracking system (ANY-maze, Stoelting). The percentage of time spent in the open and closed areas was used as measures of anxiety-like behavior, with larger time in the open arms indicating lower levels of anxiety.

### Open field

The open field test was conducted to assess animals' general locomotor activity, exploratory behavior, and anxiety-like behavior at 4 and 18-19 months of age. The open field apparatus (Stoelting) consisted of a square open field (40 cm × 40 cm) surrounded by opaque walls (35 cm high) and was dimly lit with a single light bulb directly above the apparatus, which illuminated the arena at approximately 5 lx in the center and 9 lx in corners. Each mouse was placed in the center of the open field box and allowed to explore the box freely for 10 min. The total distance traveled and the number of entries into, and the time spent in, the center of the arena (20 cm × 20 cm) were recorded with the ANY-maze video tracking system. This was repeated for three consecutive days to assess how animals would habituate to the increasingly familiar environment.

### Morris water maze

Mice were tested in the Morris water maze for spatial reference memory at 4, 7-8, and 12 months and for spatial working memory at 15 months of age. The water maze consisted of a circular tank (120 cm in diameter) filled with water made opaque with nontoxic white paint and maintained slightly above the room temperature (25 ± 2°C). All water maze tasks involved the animal finding a circular platform (10 cm in diameter) submerged in the water in order to escape from the water. On each trial, the mouse was released into the water facing the wall of the pool and allowed to swim freely in the pool to find the platform for the maximum of 60 s. Once the animal located the platform, it was allowed to stay on it for 15 s. Mice that did not locate the platform within 60 s were guided to the platform and allowed to stay on it for 15 s. After 15 s on the platform, the animals were removed from the pool, gently dried with paper towels, and placed in a single holding cage under a heat lamp until the next trial. A video tracking system (HVS 2020 and 2014; HVS Image, Mountain View, CA) was used to measure parameters such as the distance traveled, escape latency, swimming speed, the percentage of time spent in the quadrants, the number of counter crossings, and the average proximity to the platform location. The experimenter's position was maintained at the southeast (SE) corner of the room far from the tank for all the water maze tasks conducted at different ages. The experimenter was visible to the animal but remained stationary.

### Visible platform task

At each age that mice were tested in the water maze, either before (4 and 7-8 months) or after (12, 15 and 18-19 months) the memory task, a visible platform task, in which the platform was made visible by attaching a small flag (7 cm × 5 cm) to it, was conducted to examine whether mice had any visual, motor, or motivational deficits at that particular age. At 4, 7-8, 12, and 15 months of age, two or three daily sessions, with three or four trials a day, were given, in which both the platform location and the starting position were changed in a semi-random manner between trials to ensure that the animal was using the proximal cue (i.e., flag) to locate the platform. At 18-19 months, two three-trial sessions were given in a single day. The distance traveled to the platform (path length) and swimming speed were measured by the HVS video tracking system. The data collected from the last session were used for data analysis.

### Reference memory task

The Morris water maze hidden platform task was performed to assess spatial reference memory at 4, 7-8, and 12 months of age. In this task, the platform was hidden 1 cm below the water level, and distal visual cues were placed on the walls surrounding the pool. The location of the hidden platform remained constant across the acquisition sessions, while the starting position was varied in a pseudo-random manner between trials within each session. The distance traveled by the mouse to reach the platform was recorded by the HVS video tracking system. At 4 months, mice received two daily sessions of three trials each with an inter-trial interval of 6-10 min and an inter-session interval of 3 h for six consecutive days. The platform was located at the center of the fourth quadrant between the center and the northwest (NW). Following a probe trial given two days after the last acquisition session of this initial reference memory task, mice received a reversal learning task for another six days, in which the location of the hidden platform was moved to the quadrant opposite to the original target quadrant (i.e., the first quadrant). At 7-8 months, the number of daily trials was reduced to three, with an inter-trial interval of 6-10 min, and the task was run for five consecutive days. The platform was placed at the center of the second quadrant between the center and the southeast (SE). Mice were tested again at 12 months and received two trials per day with an inter-trial interval of 6-10 min for eight consecutive days. The platform position was at the center of the first quadrant between the center and the northeast (NE). Two days after the last acquisition session, a single probe trial was given, during which the platform was removed from the pool, and each mouse was released from the quadrant opposite to the trained platform location and allowed to search the pool for 60 s (4 and 7-8 months) or 30 s (12 months). The time spent in the target quadrant, where the platform had been located prior to its removal, the number of crossings of a circular area encompassing the original platform location (counter: 2 × platform diameter), and the average proximity to the former platform location were measured to assess the animal's spatial reference memory for the location of the hidden platform.

### Working memory task

Mice were tested in the Morris water maze for working memory at 15 months of age. In the working memory task, the position of the hidden platform varied from day to day but remained in the same place throughout the trials of a given day. The starting position was pseudo-randomly changed from trial to trial within a given day. On each day, four trials (1 cued trial+ 3 test trials) were given. On the cued trial, each mouse was placed on the platform for 20s, after which it was removed from the platform to a single holding cage, where it remained for 5 min. After 5 min, three test trials were given, with an inter-trial interval of 6-8 min. On each test trial, the animal was allowed to swim freely to find the hidden platform for the maximum of 60 s. Any mouse not locating the platform within 60 s was guided to the platform and allowed to stay on it for 15 s. Mice were tested for 10 days, with a one-day break after the seventh day. The distance traveled for each mouse was averaged over the last three days of testing and used for statistical analysis.

### Radial arm water maze

Mice were tested in the radial arm water maze for spatial working memory at 7-8 and 18-19 months of age. An eight-arm radial arm water maze (each arm 8 cm in width and 38 cm in length) was used at 7-8 months. A six-armed radial arm water maze (each arm 20 cm in width and 30 cm in length) was used at 18-19 moths. The radial maze was placed into the same water tank as used for the Morris water maze, filled with opaque water (25 ± 2°C). The height of the walls of the maze was 8 cm above the water level. Distal visual cues were placed on the walls of the testing room. A clear submerged platform (square, 8 cm × 8 cm for the 8-arm maze; circular, 10 cm in diameter for the 6-arm maze) was placed at the end of one of the arms. In the working memory task, the platform would remain in the same arm for all trials on a given day but its location was changed pseudo-randomly from day to day. On each trial (maximum time 60 s), the mouse was released from one of the non-goal arms and allowed to swim freely to locate the platform. The release arm was pseudo-randomly changed from trial to trial. A mouse was charged with one error each time it entered an arm other than the goal arm or did not enter any arm for 15 s. The trial continued for 60 s or until the mouse ascended the platform. If a mouse did not locate the platform within 60 s, it was guided to the platform. The mouse was removed after 15 seconds on the platform. After three acquisition trials (inter-trial interval 6-8 min), the mouse was placed in a single holding cage for 30 min, after which it was given a fourth retention trial. The error scores for each mouse was averaged over the last three days of testing and used for statistical analysis. Mice were tested until the mean number of errors over three days for WT mice reached performance criteria (2.5 on Trial 3).

### Y-maze

Mice were tested in the Y-maze for spatial recognition memory and spatial working memory at 18-19 months. The Y-maze consisted of three arms of equal length (35 cm) and width (5 cm), which were interconnected at 120° and enclosed by walls (10 cm high). The inside of the arms were identical, providing no intra-maze cues. The maze was placed under a bright fluorescent light and was surrounded by distal visual cues.

### Two-trial test

The two-trial test test was conducted to assess short-term spatial recognition memory at 18-19 months. During the first trial (training trial), one of the arms of the maze was blocked, and mice were placed into one of the remaining arms of the maze (start arm) and allowed to explore the unblocked two arms for 10 min. After a 1-hr inter-trial interval, the blocked arm was opened (novel arm), and mice were placed in the start arm and allowed to explore freely all three arms of the maze for 5 min (test trial). The number of entries into and the amount of time spent in each arm were registered by the ANY-maze video tracking system. The relative position of the novel vs. known arms (i.e., left or right) was counterbalanced within each genotype to reduce place preference effects. This test takes advantage of the innate tendency of mice to explore novel unexplored areas (e.g., the previously blocked arm). Mice with intact recognition memory will prefer to explore a novel arm over the familiar arms, whereas mice with impaired spatial memory will enter all arms randomly.

### Spontaneous alternation test

Ten to thirteen days after the two-trial test, the spontaneous alternation Y-mazetest was conducted to assess spatial working memory. Mice were released to the center of the Y maze with all three arms open and allowed to explore freely for 5 min. The number and the sequence of arm entries were recorded by a video tracking system (ANY-maze). The dependent variables were activity, defined as the number of arms entered, and percent alternation, which was calculated as the number of alternations (entries into three different arms consecutively) divided by the total possible alternations (i.e., the number of arms entered minus 2) and multiplied by 100. For efficient alternation, mice need to use working memory to maintain an ongoing record of most recently visited arms, and a mouse with impaired working memory cannot remember which arm it has just visited and shows decreased spontaneous alternation accordingly.

### Fear conditioning

Mice were tested for contextual and cued fear memory at 18-19 months. Fear conditioning was conducted in a mouse conditioning chamber (18 cm × 20 cm × 28 cm) with a metal grid floor, lit with a single house light and enclosed within a sound-attenuating cubicle (Coulbourn Instruments, Whitehall, PA). The floor grid was connected to a shocker (Coulbourn Instruments) for the delivery of an electric foot shock, which was to be used as an unconditioned stimulus (US). The chamber was also equipped with a speaker connected to an amplifier for the delivery of a pure tone (2.8 kHz, 85 dB), which served as a conditioned stimulus (CS) for cued fear conditioning. The same conditioning chamber was used for testing for contextual fear memory, while, in testing for cued fear memory, the chamber was altered with a rectangular partition placed at a diagonal, wall and floor covers with novel texture, and a novel (vanilla) scent. On the first day, mice were individually placed in the conditioning chamber, and the house light was immediately turned on. One hundred and twenty seconds later, animals were presented with a continuous tone for 30 s, at the end of which an electric shock (0.6 mA) was delivered through the floor grid for 2 s and co-terminated with the tone. Mice remained in the chamber for another 30s before being removed to the home cage. Approximately 24 hr after the training session, animals were tested for contextual fear memory. The mouse was placed in the same conditioning chamber as had been used for training and observed for freezing behavior in the absence of any shock or tone for 5 min. The last 3-min period constituted testing for contextual fear memory. Approximately 24 hr after the test for contextual fear memory, mice were tested for cued fear memory. The animal was placed in the modified chamber (novel environment) and observed for freezing behavior for 2 min. After 2 min, the animal was presented with the tone CS continuously for 3 min, during which time it was again observed for freezing behavior. The last 3-min period with the tone presentation constituted testing for cued fear memory. In both tests, each animal's movements were recorded and the percentage of freezing was calculated by FreezeFrame software (Coulbourn Instruments).

At the completion of the test, mice were assessed for possible genotype effects on shock sensitivity. A sequence of single foot shocks was delivered to animals placed in the same chamber used for fear conditioning at four intensity levels (0.1, 0.2, 0.4, and 0.6 mV) in the ascending order. There were two presentations at each shock intensity level, with a 20-s inter-stimulus interval. At each intensity level, the animal's behavior was evaluated using the following scale to determine the threshold to each of these behavioral responses (0=no response, 1=ambulation, 2=flinch, 3=hop, 4=run, and 5=jump).

### Statistical analysis

Statistical analysis of most data was performed by analysis of variance (ANOVA), with one between-subjects factor (genotype) and, when appropriate, a within-subjects factor (e.g., day). When significant effects were found, the data were further analyzed by *post hoc* comparison tests (Tukey's, Sidak's, Dunnett's, or Fisher's LSD). The level of significance was set at *p* < 0.05. Statistical analyses were carried out using the Prism software (GraphPad, La Jolla, CA).
